# Supplementary material for: Effect of Air Classification and Enzymatic and Microbial Bioprocessing on Defatted Durum Wheat Germ: Characterization and Use as Bread Ingredient
Source: Foods. 2024 Jun 20;13(12):1953. doi: 10.3390/foods13121953 (PMC11203247; doi:10.3390/foods13121953)
Supplement: Supplementary file 1 [file foods-13-01953-s001.zip › foods-3040585-supplementary.pdf]

Supplementary material

Effect of Air Classification and Enzymatic and Microbial Bioprocessing on Defatted Durum Wheat Germ: Characterization and Use as Bread Ingredient

Angela Longo [1], Gianfranco Amendolagine [2], Marcello Greco Miani [2], Carlo Giuseppe Rizzello [1] and Michela Verni [1,*]

[1] Department of Environmental Biology, "Sapienza" University of Rome, Piazzale Aldo Moro 5, 00185, Rome, Italy
[2] Casillo Next Gen Food s.r.l, Via Sant'Elia, SNC, 70033, Corato, Bari, Italy
* Correspondence: michela.verni@uniroma1.it (M.V.); +39 06 4991 2395

Table S1. Particle size distribution of coarse (HF) and fine (LF) defatted durum wheat germ fractions. D-values represent the diameter (μm) at which 10, 50, and 90% of the sample mass are comprised of smaller particles.

	HF	LF
**Particle size distribution**		
**D10**	28.64 ± 0.69^{a}	2.33 ± 0.00^{b}
**D50**	166.05 ± 4.13^{a}	10.27 ± 0.01^{b}
**D90**	492.04 ± 3.24^{a}	68.00 ± 9.44^{b}

The data are the means of three independent experiments ± standard deviations (n = 3).
$^{a–b}$Values in the same row with different superscript letters differ significantly (P < 0.05).

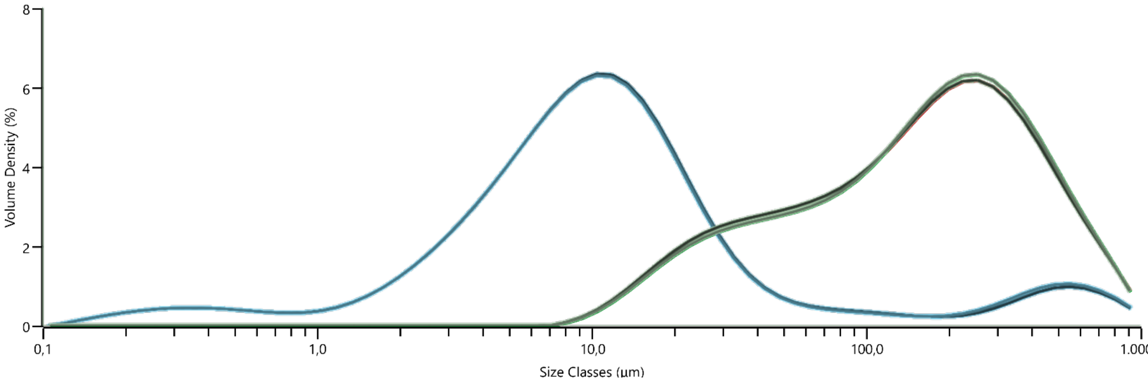

Figure S1. Graphs of particle size distribution of coarse (green) and fine (blue) defatted durum wheat germ fraction.

Table S2. Proximate composition of defatted durum wheat germ and its coarse (HF) and fine (LF) fractions.

	DWG	HF	LF
**Moisture**	7.95 ± 0.85^{b}	8.25 ± 0.35^{b}	9.15 ± 0.07^{a}
**Protein (g/100g d.m.)**	24.55 ± 1.04^{b}	17.40 ± 0.21^{c}	28.41 ± 0.57^{a}
**Fat (g/100g d.m.)**	0.88 ± 0.19^{a}	0.97 ± 0.18^{a}	0.80 ± 0.14^{a}
**Carbohydrates (g/100g d.m.)**	28.75 ± 0.55^{b}	24.55 ± 0.14^{c}	34.05 ± 0.07^{a}
**Total dietary fiber (g/100g d.m.)**	37.45 ± 1.74^{b}	49.92 ± 0.49^{a}	29.03 ± 1.48^{c}
cellulose and lignin (g/100g d.m.)	6.53 ± 0.26^{b}	10.41 ± 0.07^{a}	1.43 ± 0.42^{c}
soluble fiber (g/100g d.m.)	6.67 ± 0.39^{ab}	5.32 ± 0.42^{b}	7.82 ± 1.03^{a}
**Ash (g/100g d.m.)**	5.79 ± 0.64^{b}	4.07 ± 0.10^{c}	8.99 ± 0.12^{a}

The data are the means of three independent experiments ± standard deviations (n = 3).
$^{a–b}$Values in the same row with different superscript letters differ significantly (P < 0.05).
d.m.: on dry matter basis.

Table S3. Proximate composition of breads. WB, control wheat bread. HF-B and LF-B, control breads containing coarse (HF) and fine (LF) defatted wheat germ fractions before and after fermentation with *L. plantarum* T6B10 and *F. sanfranciscensis* A2S5 alone (f-HF-B and f-LF-B) or in combination with xylanase (xf-HF-B and xf-LF-B).

	WB	HF-B	f-HF-B	xf-HF-B	LF-B	f-LF-B	xf-LF-B
**Moisture (g/100 g)**	34.26 ± 1.41[a]	33.65 ± 1.72[a]	35.14 ± 1.81[a]	36.06 ± 1.81[a]	33.76 ± 1.01[a]	35.51 ± 1.33[a]	35.97 ± 2.02[a]
**Carbohydrates (g/100 g)**	51.80 ± 2.27[a]	49.76 ± 0.62[a]	46.32 ± 1.54[b]	47.31 ± 1.83[ab]	50.15 ±0.62[a]	47.87 ± 2.08[a]	48.25 ± 1.67[ab]
**Protein (g/100 g)**	7.56 ± 0.10[c]	7.86 ± 0.25[b]	8.15 ± 0.31[ab]	8.19 ± 0.39[ab]	8.32 ± 0.32[a]	8.45 ± 0.31[a]	8.68 ± 0.31[a]
**Fat (g/100 g)**	1.54 ± 0.09[a]	1.49 ± 0.08[a]	1.50 ± 0.01[a]	1.50 ± 0.07[a]	1.48 ± 0.08[a]	1.49 ± 0.05[a]	1.49 ± 0.06[a]
**Total dietary fiber (g/100 g)**	1.56 ± 0.11[c]	3.71 ± 0.27[a]	3.33 ± 0.31[a]	3.39 ± 0.21[a]	2.65 ± 0.17[b]	2.49 ± 0.21[b]	2.53 ± 0.26[b]
**Ash (g/100 g)**	2.28 ± 0.22[a]	2.39 ± 0.28[a]	2.45 ± 0.21[a]	2.46 ± 0.32[a]	2.59 ± 0.33[a]	2.61 ± 0.31[a]	2.55 ± 0.25[a]
**Energy Value (kcal/100 g)**	254 ± 10[a]	250 ± 11[a]	251 ± 11[a]	252 ± 8[a]	252 ± 10[a]	253 ± 9[a]	252 ± 10[a]

The data are the means of three independent experiments ± standard deviations (n = 3).

[a–c]Values in the same row with different superscript letters differ significantly ($P < 0.05$).

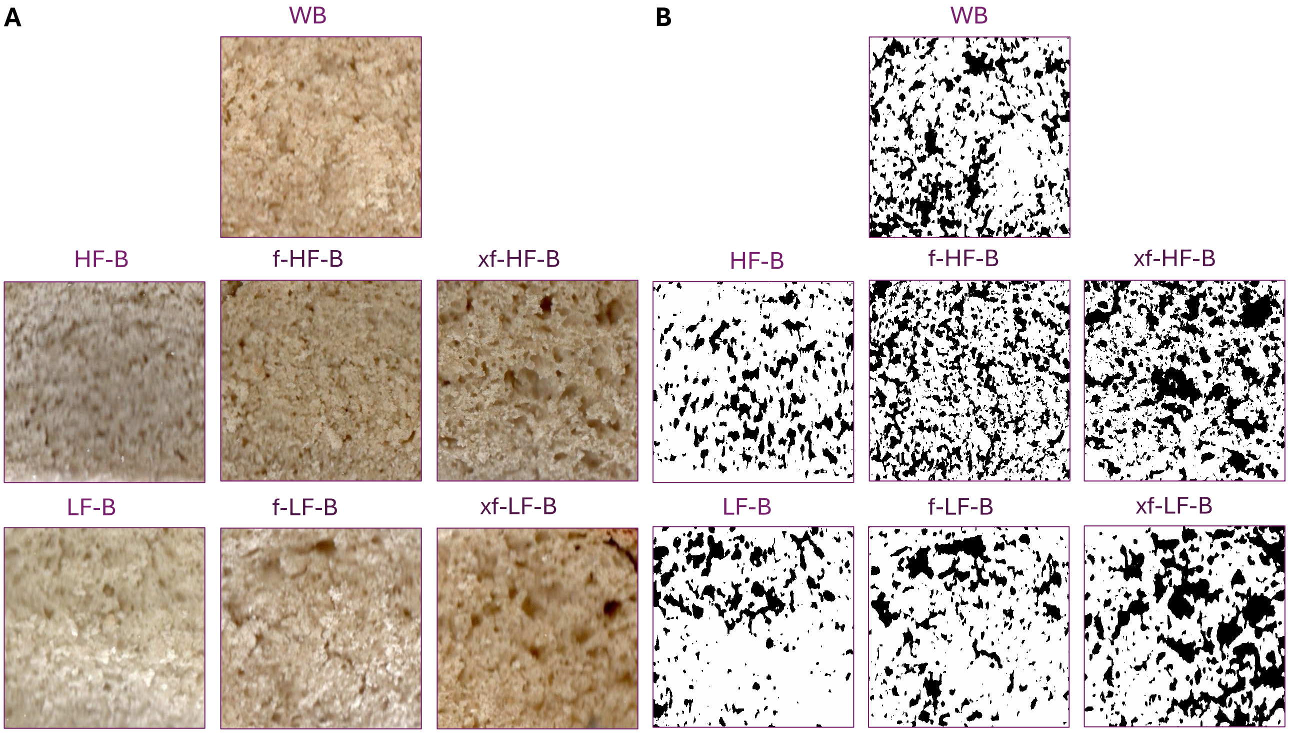

Figure S2. Digital images of breads showing the original acquisition (A) and computed binary results from gray level thresholding at the two-cluster (B). WB, control wheat bread. HF-B and LF-B, control breads containing coarse (HF) and fine (LF) defatted wheat germ fractions before and after fermentation with *L. plantarum* T6B10 and *F. sanfranciscensis* A2S5 alone (f-HF-B and f-LF-B) or in combination with xylanase (xf-HF-B and xf-LF-B).

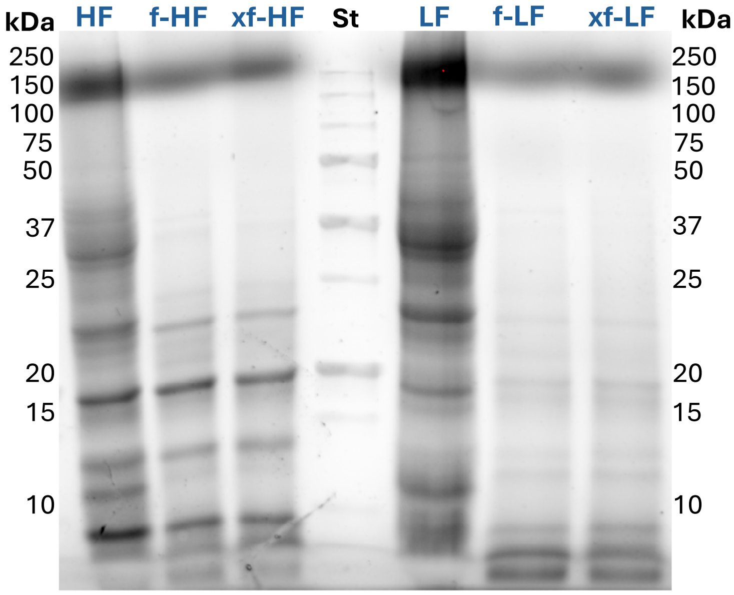

Figure S3. Profile of total proteins obtained by sodium dodecyl sulfate–polyacrylamide gel electrophoresis (SDS-PAGE) of coarse (HF) and fine (LF) defatted wheat germ fractions, before and after fermentation with *L. plantarum* T6B10 and *F. sanfranciscensis* A2S5 alone (f) or in combination with xylanase (xf). St, protein standard (Bio-Rad, Hercules, CA, United States).
